# A Dissertation on the Remote and Proximate Causes of Phthisis Pulmonalis

**Published:** 1831

**Authors:** 


ART. X.—*A Dissertation on the Remote and Proximate Causes of Phthisis Pulmonalis*—to which the Prize was adjudged for the year 1825, by the New-York State Medical Society. By ANDREW HAMMERSLY, M. D. New-York, Bliss and White, 1827.

We are led to notice the work now before us, not less by the merits of the essay and the importance of the subject, than by a wish to lead the attention of our readers to the efforts of native genius, and to direct them to the occasion which led to this publication, as an arrangement calculated to exert a very beneficial influence upon the profession.

The certain fatality which attends this disease, and the frequency of its occurrence, renders it a subject of peculiar interest. Its occurrence also is more frequent as population advances, and society increases in wealth and refinement. By an examination of the London bills of mortality, Dr. Willan has ascertained, that both the absolute and the relative mortality from consumption, has increased during the last century, though its relative proportion seems to have been considerably less at the commencement of that period, than it was fifty years before. There is reason to believe, according to Dr. Willan, that the general mortality of Great Britain has for many years been declining, but in the mean time, as the proportionate number of deaths has been declining, it admits of a question, whether the mortality arising from this cause, has not kept pace with the increase of the population.

From some of the printed lists of mortality in the town of Portsmouth, collected by Dr. Spalding, from 1802 to 1811, a period of eight years, it appears, that of 938 deaths, 199 were from consumption. Dr. S. has proved, that as great a degree of mortality arises from consumption, in the New-England states, as from malignant fevers in the Carolinas and Georgia. In London, between four and five thousand persons die annually of this disease; and throughout Great Britain, it is asserted by Woolcomb, as the result of well-founded calculation, not less than 50,000. It is to be borne in mind, however, that in consequence of the loose method of making out bills of mortality, many cases are here reported as cases of consumption, which the more accurate modern nosology, would reject as cases of ulceration of the lungs and alteration of their texture, in consequence of repeated catarrhs and pneumonic inflammation.

After some general remarks on the frequency and fatality of the disease, the author goes on to inquire into its remote and exciting causes The exciting causes are tubercles in the lungs—the predisposing, whatever tends to the development of these. The remote causes the author divides again into those which stand connected with general atmospheric conditions, and those which depend upon individual circumstances—occupation—mode of life—constitutional predisposition.

The author remarks, that those countries which are noted for sudden and violent changes of weather, are most subject to the various grades of catarrhal affection, and confirmed consumption. To this cause of pulmonary disease, the inhabitants of the northern parts of the United States are peculiarly exposed. Beside the variableness of the climate, the extremes of heat and cold in the United States, are much greater than in the corresponding latitudes in Europe—both the heat in summer and the cold in winter, being greater. Thus from a table furnished by Mr. Volney, it appears that the difference throughout the year, at Salem, is 119 degrees, while at Rome it is only 54, at Marseilles, 65, and at Padua, 87.

Certain cases in the animal economy tend to render the system more susceptible to these atmospheric causes of disease. The injury, therefore, is not proportionate to the difference between the heat of the system and the cold to which it is exposed.

"The state of the body in relation to its susceptibility of being affected by cold media, has more reference to the *kind* than degree of previously existing heat, or, more correctly speaking, although an equal quantity of actual heat may be present in the system, such heat may be abstracted with greater or less facility and safety, according to the mode in which it has been generated.

The increase of temperature, occasioned by what is termed an inflammatory action pervaving the whole system, such as is sometimes observed in inflammation of the lungs; that attended with an extremely debilitated state of the vital power, as in instances of what has been improperly termed putrid fever; the heat consequent upon violent exercise; and that produced by communication from without, as in the the example of hot baths, or exposures to other sources of great heat, while the body continues inactive, are all essentially different in their nature, and although in each case the quantity may be equal, and a thermometer applied to any part of the body's surface shall indicate the same; yet from such temperature alone, it would be improper to form a judgment, on the expediency and safety of the sudden applicatian of cold."

In many cases, the injury would appear to rise, not from the "sudden abstraction of caloric, but from the precipitated interruption of those *actions* by which the increase of temperature had been generated;" and we think, (notwithstanding the reasoning of our author,) of those actions intended to prevent the undue accumulation of heat.

His remarks on the other predisposing causes connected with occupation and habits of life, we must necessarily pass over. We cannot, however, omit inserting the following letter from Dr. Cogan, to Dr. Beddoes, containing some remarks upon the influence the domestic economy of the Dutch may have over their liability to catarrhal complaints.

"Speaking of the mode of warming their appartments, he thus remarks—"Five or six turfs, about the shape and size of our bricks, which is the usual fuel of the country, are arranged in the form of a chimney, and a glowing coal placed at the top, by which means the inward surfaces are enkindled, and the turfs are half consumed before any share of a very moderate heat is received in the apartment. The females never approach the fire, but generally place themselves at the greatest distance, contented with a small coal of the turf completely charred in an earthen pot, filled with ashes, to moderate the heat. This is placed in a wooden box, with a perforated surface, and applied to the feet.

"Supported by this consolation, they prefer placing themselves at the greatest distance from the fire, generally by the windows, which by the way, from their immense size, greatly contribute to the coolness of the rooms. In short, by the extreme airiness of their rooms, and the warmth of their dress, they are secured against those extremes of heat and cold, to which the inhabitants of those countries are hourly exposed during the winter season. Their customs are as direct contrary to ours, it being customary among us to dress as lightly as possible, and rer der our apartments as warm as possible, by the united aid of large coal fires, double doors, warm carpets, and ceiled rooms, and by every caution that can prevent the external air from entering at chinks and crevices, to restore the balance of circulation. This contrariety in the mode of living, in these two essential articles of dress and habitation, will fully explain, my dear sir, the cause of the frequency of catarrhs in this country, and their being comparatively seldom in Holland, without imputing it, exclusively, or principally, as s me have done, to the general variableness of our climate. The transitions from heat to cold in Holland, are fully as frequent as in England, and the extremes of heat and cold are generally greater; but their effects upon the constitution are by no means so immoderate or violent. Thus, I fear, that the opprobrium that has been cast upon the climate of England, rather belongs to the injudicious conduct of its inhabitants. It has been remarked, that as luxury increases in Holland, respecting the greater comfort and accommodations of their apartments, they are becoming more subject to catarrhs."

It is unnecessary to follow the author through his detail of the opinion of nosologists respecting this disease. In this, as in many other instances, the errors of our ancestors have no c laim to the prominent station which almost all writers think themselves bound to assign them. The author has adopted the views of Laennec, and embodied his opinion of the disease in the following propositions:

"1st. Phthisis pulmonalis has claims to be considered as an idiopathic disease. 2d. The previous existence of inflammatory symptoms, is not necessary to the developement of phthisis pulmonalis, but that such as are characteristic of irritation, existing in a greater or less degree, do commonly occur. 3d. Tuberculous formations are the real proximate cause of this disease.'

Under the first of these heads, in combatting the opinion of Dr. Cullen, that this disease is a sequel of hemoptysis, Dr. H. makes the following just remark. Constitutional irritability is a forerunner of both phthisis and hemoptysis, but in the former it arises from the weak condition of the lymphatic tissue, while in the latter it arises from, or is connected with the general rapidity of the circulation. The pulse also in the former, invariably rapid, wants the tension and fulness which attends the latter in its forming stage, and the debility which attends its latter stages.

The question relating to the previous existence of inflammation, is supposed to be intimately connected with that of its scrofulous origin. Many have been unwilling to assign the character to phthisis, from the difficulty of detecting lymphatics or glands in the lungs. But if there were no difference of opinion among anatomists upon this subject, and we were prepared to give up the glandular structure of the lungs, still the general habit of the subjects of tubercular consumption, and the analogy between the tubercles in the different stages of their progress, and other scrofulous affections, would sufficiently establish this question. In addition to this, the author remarks, that the disease is most prevalent under those circumstances which are particularly favorable to scrofula. Admitting this analogy, we will be prepared still further to concede, that as scrofula frequently occurs without previous inflammation, tubercles may in like manner be formed. This view is strengthened by their simultaneous appearance in different parts of the body, where no inflammatory symptoms whatever have been observed. The fact, in our estimation, appears to be, that both scrofulous and tuberculous engorgements, in their forming stage, imply the very opposite of inflammation; and that when fully formed, a degree of irritation so slight as to pass unnoticed, is sufficient to bring on the stage of ulceration.

While we do not deny that consumption may follow, as the consequence of abscess, produced by pneumonia, we believe it very rarely appears in that way, and concur, fully, in the sentiments advanced by Dr. H., in the following quotation:

"We do not hesitate to declare it, as our unreserved and candid opinion, that, in many instances, the medical adviser will forewarn, very seriously, those concerned in the welfare of his patient, of the danger of approaching consumption, unless the severity of present symptoms should subside; when lo! the enemy has already entered the frail fabric of his victim, and is probably too firmly entrenched to be dislodged by the combined powers of skill and experience. But, perhaps, he has waited for the developement of inflammatory symptoms, and stands ready, with lancet in hand, to meet their indications; if so, we fear he has often sacrificed the lives of those committed to his trust, in compliance with the dogmas of an insecure faith, or the prejudice of long cherished opinions. Had he exchanged the maxims of the closet for the benefit of personal examination, and made his own deceased patients the subject of his instruction, he would often have found the tuberculous lung, without any of the appearances which denote previous inflammation, and been taught a lesson of lasting utility. The error of such writers as have made consumption a constant sequel of pneumonia, will we imagine, be sufficiently apparent, from what we have advanced on this topic. Those who have gone so far as to deem preumonia the proximate cause of the disease, thereby disregarding the important influence of the tuberculous formations, have most certainly mistaken the true theory of the subject."

We cannot better conclude this article than by giving the following extract from Laennec, whose patient and laborious investigations have done more to advance our knowledge of the pathology of this disease, than the speculations of all the writers that have preceded him. Speaking of tubercles, he says:

"Those bodies, when first observable in the substance of the lungs, have the appearance of small transparent grains, greyish or colorless, and varying from the size of a millet seed, to that of a hemp seed. In this their first state, they may be called *miliary* tubercles. These gradually increase in size, and become yellowish and opaque, at first in the centre, and, successively, throughout their whole substance. In their progressive and natural increase, several unite together, so as to form larger masses of the same kind, which, like the individual ones, are of a pale yellow opaque, and of the consistence of very firm cheese. In this stage, they may be named *crude* or *immature* tubercles. It is in this stage of their progress, that the substance of the lungs, which had been hitherto healthy, begins now to grow hard, greyish, and semi-transparent round the tubercles, by means of a fresh production, and seeming infiltration of tuberculous matter in its first or transparent stage into the pulmonary tissue. It also sometimes happens, that considerable portions of pulmonary tissue put on this character without any previous developement of individual tubercles. Parts so affected are dense, humid, quite impermeable to air, and exhibit, when cut into, a smooth and polished surface. Gradually there are developed in these comparatively solid and pelucid masses, an infinity of very minute, yellowish, opaque points, which, increasing in size and number, at length convert the whole diseased space into a tuberculous mass, of the kind named crude, or immature. In whatever mode the tubercles have first shown themselves, they at length, after a very uncertain period, become first softened, and finally liquefied. This change of consistence commences in the centre, and progressively approaches the circumference. In this stage the tuberculous matter is of two different kinds in appearance, the one resembling thick pus, but without smell, and yellower than the immature tubercle; the other a mixed fluid, one portion of it being very liquid, more or less transparent and colorless, less tinged with blood, and the other portion opaque, of a caseous consistence, soft and friable. In this last condition, which is chiefly observable in strumous subjects, the fluid perfectly resembles whey, having smaller portions of curd floating in it. When the softening of the tuberculous mass is completed, this finds its way into some of the neighboring bronchial tubes, and as the opening is smaller than the diseased cavity, both it and the latter remain of necessity fistulous, even after the complete evacuation of the tuberculous matter. It is extremely rare to find only one such excavation in the tuberculous lung. Most commonly, the cavity is surrounded by tubercles in different stages of their progress, which, as they successively soften, discharge their contents into it, and thus gradually form those irregular and continuous excavations so frequently observable, and which sometimes extend from one extremity of the lungs to the other.." "The above is the ordinary mode in which tubercles are developed; but there are two other modes, which, although mere varieties of the former, are yet deserving notice. The one is, where, in a lung containing tubercles in different stages, we find small portions of the pulmonary tissue, seemingly infiltrated by a gelatinous-looking matter, of a consistence intermediate between liquid and solid, transparent, and of a light greyish or sanguineous hue. In these diseased portions, the cellular structure of the lung is quite destroyed, but we can perceive in them a multitude of very small points, of a yellowish white color, and opaque, and which are evidently portions of the tuburculous matter, which has reached the second stage of its progress, without there being any surrounding portions of the greyish substance, which denotes the first stage.

The second mode of anomalous development of tubercles, appears likewise to take place, without any previous formation of gray matter; at least, if there be such, the transition from it to the second stage is so rapid, that I have never been able to detect its presence. In this variety, we find here and there, in the lung, tuberculous masses of a yellowish-white color, much paler, less clear, and differing less from the substance of the lung, than the ordinary immature tubercle. These masses are irregular, angular and have scarcely ever the rounded forms of ordinary tubercles. They seem, like the variety described in the preceding paragraph, and like the diffused, gray matter, noticed before, to be an infiltration of tuberculous matter into the pulmonary tissue, while the proper, or rounded tubercles, are foreign bodies, which separate or press it aside, rather than penetrate it. These masses may, therefore, properly enough be named, tubercular infiltration of the lungs. They occupy sometimes a considerable portion of one lobe. When they reach the surface, they occasion no prominence on the part, nor in any degree alter its form. As they increase, they assume the yellow color of the tubercles, and terminate by softening in the same manner. These three varieties of tuberculous degeneration are often found in the same lung. Sometimes I have found this last variety alone, in lungs affected with peripneumony, and this even in the hepatised portions. In these cases, the small number and extent of the diseased masses, and their deep pale color, showed their formation to be recent."

